# Editorial: Advances in the care of the pediatric pulmonary hypertension patient: from the neonate to the adolescent-young adult patient

**DOI:** 10.3389/fped.2023.1220070

**Published:** 2023-05-25

**Authors:** Catherine M. Avitabile, Rachel K. Hopper, Stephanie S. Handler, Angela Bates

**Affiliations:** ^1^ Department of Pediatrics, University of Pennsylvania Perelman School of Medicine, Division of Cardiology, Children's Hospital of Philadelphia, Philadelphia, PA, United States; ^2^ Department of Pediatrics, Stanford University School of Medicine, Division of Cardiology, Lucile Packard Children's Hospital, Palo Alto, CA, United States; ^3^ Department of Pediatrics, Medical College of Wisconsin, Division of Cardiology, Children's Hospital of Wisconsin, Madison, WI, United States; ^4^ Department of Pediatrics, University of Alberta, Division of Cardiology Stollery Children's Hospital, Edmonton, AB, Canada

**Keywords:** pediatric pulmonary hypertension, bronchopulmonary dysplasia, risk assesment, pulmonary vasodilator medications, exercise, clinical trials

**Editorial on the Research Topic** Advances in the care of the pediatric pulmonary hypertension patient: from the neonate to the adolescent-young adult patient

In pediatric pulmonary hypertension (PH), occlusion of small pulmonary arteries leads to increased right ventricular afterload and risk of right heart failure. In recent years, therapeutic options have increased for young patients, however, in the United States most treatments are given “off-label” without formal approval from the Food & Drug Administration. Despite treatment advances, PH remains a life-altering and life-limiting diagnosis for many infants and children. This Research Topic presents novel approaches to the diagnosis, treatment, and risk assessment of PH across the pediatric spectrum. Contributions address novel medical and transcatheter interventions, multidisciplinary care, and risk scoring in pediatric PH. The collection of manuscripts increases awareness of the complexity of this disease and advances the goal of improving functional status, quality of life, and survival in children with this serious condition.

Several manuscripts present critically important pediatric experience with off-label pulmonary vasodilator therapies. Tadalafil is a once-daily, long-acting phosphodiesterase type 5 inhibitor with a favorable side effect profile but limited data in children. Youssef and colleagues describe improvement in right ventricular function and minimal side effects in 154 children treated with tadalafil suspension (both *de novo* and transition cases) at two North American centers. These encouraging data are especially relevant in young children with PH who cannot tolerate enteral tablets due to age, developmental status, or feeding tube requirements. Domingo and colleagues describe novel use of riociguat, an oral soluble guanylate cyclase stimulator, in 2 infants with genetic mutations and severe, nitric oxide-dependent pulmonary arterial hypertension who did not respond to sildenafil. This experience should prompt further study of riociguat, especially in neonatal populations with respiratory failure and high utilization of nitric oxide, in order to expand therapeutic options in the sickest children. Three manuscripts add to knowledge about selexipag, an oral selective prostacyclin receptor agonist, with clear benefits in adult PH but limited published experience in pediatric PH. In a case report, Hasan and colleagues describe a neonate with developmental lung disease related-PH refractory to phosphodiesterase type 5 inhibitor and endothelin receptor antagonist treatment who demonstrated clinical and echocardiographic improvement with selexipag. In a second manuscript, Youssef's group describes a heterogeneous group of 24 PH patients treated with selexipag at 3 Canadian centers. In short term follow-up (12 months), most patients maintained clinical stability with expected gastrointestinal side effects. Faircloth and colleagues describe their experience initiating, transitioning, and titrating selexipag in 7 children (2 *de novo*, 5 transition from continuous prostacyclin) younger than 10 years of age with weights 10–30 kg. Their institution-specific algorithm provides practical insight for other centers and supports continued successful use in the pediatric population. These studies also highlight the reliance of pediatric PH treatment on case reports and observational studies given the challenges of performing randomized clinical trials in children. However, continued observational work and novel trial designs are critical to test pulmonary vasodilator therapies in the pediatric population and increase access to medications traditionally limited to adult use.

Two manuscripts describe innovative transcatheter interventions to treat pediatric PH. Closure of an atrial septal defect can be a challenging decision in pediatric PH, particularly in those with Group 3 lung disease related-PH as providers may weigh the benefit of alleviating arterial damage from left-to-right intracardiac shunt against the loss of the right-to-left “pop off” during times of acute RV failure or low cardiac output. In a third manuscript, Youssef and colleagues report a single-center, Canadian experience with atrial flow regulators and fenestrated atrial septal defect occluders, allowing controlled closure of these defects with some degree of right-to-left shunt if needed. The data suggests Group 1 patients may demonstrate clinical improvement with this innovative approach, and future multicenter studies should identify ideal pediatric candidates and optimal intervention timing. Additionally, Haddad and colleagues describe their experience with transcatheter Potts shunt creation in children with severe pulmonary arterial hypertension. The reverse Potts shunt creates or maintains a connection between the pulmonary artery and aorta, allowing right to left interarterial shunting and prevention of right ventricular failure at the expense of lower extremity cyanosis. In Haddad's report, 13 patients underwent endovascular stenting of a patent arterial ducts or aorta-to-pulmonary radiofrequency perforation and covered stent placement. At median follow-up of 77 months, patients demonstrated improvement in functional class and high 6-year survival (92.3%) with frequent transcatheter re-intervention on the graft or stent. These data further inform our understanding of the longer-term outcomes of this palliative alternative for patients with severe PH and exhaustion of treatment options other than lung transplantation.

The breadth and complexity of pediatric PH is highlighted in several manuscripts detailing a need for comprehensive, multidisciplinary care in this population. Yung and colleagues outline their center-specific, systematic approach to care of infants with bronchopulmonary dysplasia related-PH (BPD-PH). With optimization of respiratory support, low threshold for closure of intracardiac and interarterial shunts, and initiation of pulmonary vasodilators only after diagnostic confirmation by cardiac catheterization, they report universal resolution of PH. In particular, these findings emphasize the need to consider the impact of shunts in patients with developmental lung diseases and limited pulmonary vascular beds. Sullivan and colleagues also augment the understanding of BPD-PH through their description of cardiac catheterization data from 34 patients with BPD, 32% of whom had left atrial hypertension defined as a pulmonary capillary wedge pressure >10 mm Hg. Left atrial hypertension was associated with increased risk of tracheostomy and/or death, suggesting that left atrial hypertension (from left ventricular diastolic dysfunction or intracardiac shunts) may play a role in pathogenesis and patient outcomes.

Comprehensive pediatric PH care requires many subspecialists including geneticists, nutritionists, laboratory medicine specialists, rehabilitation teams, and other experts. Ishizuka and colleagues report the burden of heritable pulmonary arterial hypertension at a large PH referral center. Among 66 patients who underwent genetic testing, 14% were found to have a pathogenic mutation, most commonly BMPR2 mutations, with severe hemodynamic findings. These “real-world” single-center clinical data support larger scale population studies to characterize the burden of heritable pulmonary arterial hypertension in the pediatric population in order to develop targeted treatment approaches. Ruland and colleagues demonstrate that malnutrition is underappreciated and underdiagnosed in pediatric PH but that treatment by a registered dietitian improves malnutrition status in many patients at their center. The need for close monitoring and individualized nutrition recommendations can be delivered through a multidisciplinary team at a comprehensive pediatric PH center. Wieland and colleagues report acquired von Willebrand syndrome with bleeding complications in a series of patients with severe PH requiring bilateral lung transplantation, highlighting the importance of considering this diagnosis in patients undergoing surgical interventions or experiencing trauma. Avitabile and colleagues reported associations between skeletal muscle deficits and worse exercise performance on cardiopulmonary exercise testing and exercise cardiac magnetic resonance imaging in adolescents with PH. These data may support interventions to improve muscle mass and strength as a potential means to improve functional status and quality of life in this population.

Finally, Lokhorst and colleagues present a systematic review of risk stratification in adult and pediatric PH, which is critical to determining treatment strategy. They identified the most common variables in risk stratification models as: World Health Organization functional class, 6-minute walk test distance, N-terminal pro brain natriuretic peptide level, and mean right atrial pressure, cardiac index, and mixed venous oxygen saturation on cardiac catheterization; however, they identified very limited data on risk stratification in pediatric PH. Future studies must test these adult-focused scoring systems in large pediatric PH registry populations and identify new pediatric-specific imaging parameters, serum biomarkers, and physical activity metrics that predict mortality in children.

In conclusion, this Research Topic presents important advances in pediatric PH, but we are reminded that much work remains to be done. This collection provides a framework for identifying future areas of research for pediatricians, advanced practice providers, and PH providers who share the common goal of advancing the care of this vulnerable population from the neonatal period to adolescent-young adulthood.

We are grateful to the many pediatric PH experts who contributed their science to this Research Topic collection and to the peer reviewers and editors who provided critical feedback which further improved the quality of the publications.

